# A Description of an Artificial Palate, Velum, Uvula, Septum of the Nose, Alveolar Process and Artificial Tooth

**Published:** 1852-01

**Authors:** Joseph Linderer

**Affiliations:** Dentist, Berlin, Prussia.


### ARTICLE V.

*A Description of an Artificial Palate, Velum, Uvula, Septum of the Nose, Alveolar Process and Artificial Tooth.* By JOSEPH LINDERER, Dentist, Berlin, Prussia.

IT sometimes happens, by a fault of nature, that the whole of the roof of the mouth is wanting; so, that eating and drinking are rendered very difficult, the cavity of the mouth and nostrils becoming one, part of the food and drink received into the mouth, comes out through the nose, speech becomes almost unintelligible. For this evil, surgery can do nothing. The resources of art alone, can afford relief. I will describe a case which came under my own treatment.

A young man about twenty years of age, who was found in the Orthopedic Institute of Health, by counsellor Berend, and sent to me by him, suffered from a congenital defect of this kind. There was a fissure in the upper lip, an opening in the middle of the alveolar border, of the form of a triangle, the base of which was towards the nose. This, from anterior to posterior, was a little more than four lines, and the lower part of the opening was four and half Paris lines in breadth. The height of the opening was four lines. The teeth which ought to have been in the jaw, were partly present and partly wanting. There were on the right side, first, the lateral incisor, which was of a conical form, then the cuspid, on the point of which, erosion was noticed, then again, the two bicuspids. The grinders were entirely destroyed by caries. On the left side stood, first, the cuspid, on the point of which, erosion was also observed, then, the two bicuspids. The grinders were wanting here too, having been destroyed by caries. There were thus wanting, in a space only four and a half lines broad, two central incisors and one lateral. Besides, what has already been stated, the alveolar border, which received the two cuspid teeth and the lateral incisor, was much shorter than it ought to have been, so that the front teeth in the lower jaw stood considerably in front of the anterior part of the upper dental arch, and there was an opening in front when the molar teeth rested one upon another. The breadth of this opening was fourteen and a half lines and its height in the center, seven and a half lines. The lower incisors and cuspidati had suffered much from erosion. Back of the defect in the alveolar arch of the upper jaw, there was an opening in the arch of the palate, seven lines in breadth. The velum was also divided into two parts, each of which had at the end half of the uvula. These parts hung loosely. A palate suture could not have been used. It will be seen from this description that the cartilaginous septum of the nose was wanting. While a hole in the hard palate can always be repaired with safety, cases like the one here described, offer the most important difficulties, from the fact that the soft movable part must be imitated. The artificial appliance must be constructed in such a manner, that it may, as much as possible, make that part good. It is not possible, however, to do this perfectly, for the soft palate is not only drawn backward, but it also draws itself together. This latter seems to be of less importance.

The principal thing on which depends, then, is, that the movable part of the artificial palate, representing the velum and the uvula, first, should have the right, the accustomed position ; second, that it may be pressed upward, in the right way. Besides, the proper form must be given to it.

This last is difficult, because, in persons thus afflicted, that part, in its natural condition, is lacking, and hence, it is not easy to make a model which will correspond to the form ; besides, it is impossible to take an impression of the natural position of that part, for the reason, that it is not present. Of the movement made by the velum in swallowing, it is still less possible to take an impression. If the movable part has not the proper accustomed position, first, it cannot join itself closely to the remaining soft parts on the sides, and food or drink might come between. Speech will be rendered still more difficult, and deglutition also, because the back part of the cavity of the mouth is too low. Second, if it presses too hard on the remaining soft parts, it might excite inflammation, besides deglutition will be rendered more difficult, if the back part of the cavity of the mouth is too high. If this artificial part has not the right size, and if it, or even the uvula alone, should hang too low, it may interfere with the tongue, in which case it will be likely to excite nausea and retching. If, on the other hand, it should be too short, food might get in on the upper surface. Food and drink might also get in above, if the artificial part does not fit closely to the movements of both the existing parts of the soft palate, and speech would suffer still more.

Thus we see that there are many things here to be considered. In order to remove, as far as possible, these difficulties, it is necessary, first, that we know exactly the form of the back part of the cavity of the mouth, when at rest; second, what are the movements of the soft parts during deglutition. The description of these would be too circumstantial, and still, perhaps, not adequate, and, therefore, it is best that one who wishes to make a compensating piece for that part, should most carefully observe it in motion and at rest, first in persons in whom it is perfect, and then in those in whom it is diseased or imperfect.

*The Impression.*—The first thing to be done, is to take an impression. This must be done with the greatest possible care, since, upon it depends, to a great extent, the result. But in the present instance, this is much more difficult than usual. I will, therefore, mention what is necessary thereto. The wax impression may be the same as that used in ordinary cases, for a palate plate appertaining to it is all that stands in the way. That about which we must be particularly careful is, that the wax-holder should be sufficiently long to embrace the tuberosity at each end of the alveolar border, in its whole length and height. In other respects, also, the wax-holder must fit before it is filled, and therefore it should be tried in before it is filled with the wax. It is especially necessary that it should be sufficiently broad at the back part, the outward edges not too high, and the inward wall having an oblique direction inwardly. The wax for the impression should be sufficient in quantity to fill the wax-holder to the proper height, that a plate half an inch thick may be placed on the hindpart, and on the forepart of the palate—and that the diseased opening may still be filled to the height of half an inch. After the box has been inlaid with paper in the usual manner, it should be filled with wax, previously softened, sufficiently high that an impression of even a part of the hard palate may be taken. In the forepart, where the defect in the alveolar arch is, the wax must, of course, be much higher and indeed correspond in form. The impression of the alveolar arch must now be taken, using especial care that the tuberosity on each side shall make its impression perfectly. To this end, the wax which is forced backward must be pressed forward against the hindermost wall of the tuberosity with the forefinger of the right hand, whilst the inward free projecting wax must be firmly pressed against the palate, with the thumb of the same hand, and into the diseased opening as high as possible. We now take new wax in sufficient quantity, which should be at hand, and so soft that it may be pressed against the soft parts without troubling and without pressing them too far back. This may be put in the mouth all at once, or only a part at the time, between the wax-holder against the inner walls of which it must be firmly pressed, and against the wax already there, then against the remaining free part of the hard palate, and into the existing defective places, and last of all, against the soft parts, especially on the sides. This cannot be done with much force.

From this, three models of plaster of paris should be made. One for the procurement of two models for stamping palate plates, the second to work upon; the third will be needed if the second should be injured by being worked at. If the impression has been rightly taken, the soft part of the palate will have received the right form, except that it lies a little more above, but that is not a matter of any moment. The opening in the plaster model leading towards the nose, must, in the necessary manner, be filled with plaster, upon which the two metal models are to be made.

The palate plate for the hard palate, must be finished first, and extend so far back that the posterior edge will cover the tuberosity. The missing part of the alveolar arch anteriorly, must also be covered. The fastening is effected in the usual way, to the remaining back teeth, by side braces and clasps. The missing part of the alveolar ridge is counterfeited by a piece of whale-bone, colored red, and a tooth fastened upon it. In the case in hand, only one tooth could be fastened upon it. An imitation of the dividing cartilage of the nose is easily made. The whole must be so constructed that the palate on being put in, shall not press the sides of the mouth, and gradually force the maxillary bones apart in front, as they are only united by the upper half of the alveolar arch, so that a steady, even, though feeble pressure, might cause this evil result.

The movable part of the palate is made in the following manner. 1st. The front edge is fit exactly upon the back edge of the hard palate. 2nd. From each end of the front edge there proceeds outwardly, a process covering each side of the tuberosity of the upper jaw, and, indeed, the whole upper surface. 3d. That part of the movable plate reaching backward from the tuberosity plates is much broader than the plate for the hard palate, indeed, so broad that the whole velum on its entire natural breadth is imitated. This breadth and the side plates must be present, because, in deglutition the soft parts draw themselves strongly together and upward; if now the artificial parts were not made in the manner mentioned, food and drink would force themselves into the nose on each side of the false palate. 4th. The under back edge forms two concavities, as they are seen in the natural condition, still, it is represented without the uvula. This plate is also stamped by means of the metallic model.

Now we make the regulating rod of the velum. This last can only have, when deglutition is not taking place, a particular position in the mouth, and must not sink too far down, on the other hand, it must not rise too high during the performance of this function. I managed this in the following way. Upon the middle of the upper surface of the hard palate, and two lines from the posterior edge, I soldered two staples side by side. Between these came the regulating rod, which, at that place, is perforated and secured by means of a pin which passes through it and the staples and is riveted. The forepart of the rod is four lines long, and its end one and a third line from the plate, the back part is one inch long, round, and is run through a bar, soldered upon the middle of the upper surface of the velum, four and a half lines from the front edge. By this means, the artificial velum is prevented from sinking deeper in the mouth than it should. But, if in deglutition it should be pressed up too much, then the hinder part of the rod will lift itself up while the forepart will sink. But since this can only be done to the extent of one and a third lines, the velum, also can only be pressed so far, because the regulating rod stops it.

The securing of the parts of the plate, as also the motions of the velum, is effected by a gum-plate.

But since the uvula of a soft part must be of gum, and the edges of the velum be soft, we must, therefore, have a velum with the uvula, made of gum large enough to come forward and cover the hard artificial palate, to the breadth of one line, and reach out a little beyond the edge of the curtain.

The fastening of this gum-plate upon the metallic plates must be done with silk thread, by means of sewing. For this purpose a number of very small holes must be made through the back edge of the hard palate, and through the front edge and the other edges of the palate-veil, all, one half line from the end. The front edge of the gum-covering must first be sewed to the back edge of the hard palate, indeed, of the surface of the mouth. Then the metallic covering must be brought into the proper position to the artificial hard-palate, and first, the front edge must be sewed to the gum-plate and then the other edges, which done, the artificial palate is completed.

Since by the strong and frequent movements of the artificial palate, the front edge of the gum-plate, when it is sewed on, might be easily torn out, in which case the palate would be useless, it becomes necessary, before sewing it on, to bind the edge of it with a band of gum, which is very durable. In order that the silk employed may not too easily suffer from the saliva, it ought to be smeared with dissolved gum. We must take especial care not to have the uvula too long.

The result of this contrivance of mine in respect to eating and drinking was excellent. The patient felt not the slightest inconvenience, deglutition took place without trouble and after meals, no traces of food were to be seen on the nasal surface of the palate. Health counsellor, Berend, presented this young man to the National Society of Physicians, in whose presence he ate and drank. In regard to specch, the machine had produced no important change; in the mean time, it must be noticed, that I could only observe the patient on that particular day on which he received the palate, for on the next day he left Berlin, and, indeed, it is often the case with inserted teeth, that the speech, which at first is somewhat injured, afterwards becomes perfectly good. But if this should not result from the artificial palate, still we might remedy this defect by art, perhaps by some alteration of the machine, but at least by giving the patient instruction in regard to speech, for it is natural, that the organs which are necessary for speech, and which have been moving in this imperfect manner for so many years, should be obliged to unlearn this, and that it is possible we may well suppose.

